# *Rickettsia slovaca* Infection in Humans, Portugal

**DOI:** 10.3201/eid1910.130376

**Published:** 2013-10

**Authors:** Rita de Sousa, Branca Isabel Pereira, Claúdia Nazareth, Susana Cabral, Conceição Ventura, Pedro Crespo, Nuno Marques, Saraiva da Cunha

**Affiliations:** National Institute of Health Dr. Ricardo Jorge, Águas de Moura, Portugal (R. de Sousa);; University Hospitals of Coimbra, Coimbra, Portugal (B.I. Pereira, C. Nazareth, S. Cabral, C. Ventura, P. Crespo, N. Marques, S. da Cunha)

**Keywords:** TIBOLA, tick-borne lymphadenopathy, *Dermacentor*-borne-necrosis-erythema lymphadenopathy, DEBONEL, *Rickettsia slovaca*, lymphadenopathy, facial edema, Portugal, rickettsiae, rickettsial infections, vector-borne infections, scalp eschar, *Suggested citation for this article*: de Sousa R, Pereira BI, Nazareth C, Cabral S, Ventura C, Crespo P, et al. *Rickettsia slovaca* infection in humans, Portugal. Emerg Infect Dis [Internet]. 2013 Oct [*date cited*]. http://dx.doi.org/10.3201/eid1910.130376

## Abstract

Fifteen years after the initial detection of *Rickettsia slovaca* in ticks in Portugal, 3 autochthonous cases of *R. slovaca* infection were diagnosed in humans. All patients had an eschar on the scalp and lymphadenopathy; 2 patients had facial edema. *R. slovaca* infection was confirmed by serologic testing, culture, and PCR.

*Rickettsia slovaca* is a pathogenic, tick-borne, spotted fever group (SFG) rickettsiae that was initially isolated in 1968 from a *Dermacentor marginatus* tick in Slovakia. *R. slovaca* infection has been described in humans from several countries (*1*,*2*), but a laboratory-confirmed case of *R. slovaca* infection was first reported in a patient in France in 1997 (*3*). *R. slovaca* has since been reported in humans in France, Spain, Hungary, Slovakia, Bulgaria, Italy, and Germany (*4*–*7*). 

The analysis of a large series of patients reporting the common clinical signs of enlarged regional lymph nodes associated with the tick bite led to the names that have been used to designate this rickettsial disease, tick-borne lymphadenopathy (TIBOLA) and *Dermacentor*-borne-necrosis-erythema lymphadenopathy (*8*,*9*). Apart from Mediterranean spotted fever, TIBOLA may be among the most prevalent tick-borne rickettsioses in Europe (*4*). 

In Portugal, *R. slovaca* was initially described in 1995 in *D. marginatus* ticks and later in *D. reticulatus* ticks (*10*,*11*) but has not been identified in humans. We report 3 laboratory-confirmed cases of *R. slovaca* infection in human patients in Portugal. 

## The Study

During October 2010–May 2012, three Caucasian women who sought care at the emergency department of University Hospitals of Coimbra (HUC), Coimbra, Portugal, were admitted with clinical signs and symptoms compatible with a rickettsiosis. The patients were residents of rural areas of the Coimbra district, and all were at risk for tick exposure through fieldwork or direct contact with domestic animals (Table). Two patients reported that they had removed a tick from the scalp. 

**Table:** Epidemiologic, clinical and microbiologic characteristics of 3 patients infected with *Rickettsia slovaca*, Portugal

Characteristic	Patient 1	Patient 2	Patient 3
Epidemiologic			
Age, y/sex	50/F	53/F	30/F
Date of illness onset	2010 Oct	2012 Apr	2012 May
Type of residence	Rural	Rural	Rural
At-risk activity	Field worker	Field worker, contact with domestic animals	Contact with dogs
Reported tick bite	Yes	Yes	NA
Clinical characteristics			
Incubation time, d*†	4	7	NA
Fever	Yes, 37.8°C	No	No
Eschar on scalp	Yes	Yes	Yes
Rash	No	Yes, maculopapular	No
Painful cervical/occipital lymphadenopathy	Yes	No	Yes
Facial edema	Yes	Yes	No
Headache	Yes	No	No
Localized alopecia	No	Yes	No
Laboratory diagnostics			
Antibodies against *R. slovaca* by IFA‡			
Sample 1, titer IgM/IgG	Negative	32/64	32/64
Sample 2, titer IgM/IgG	32/128	1,024/1,024	ND
Culture from eschar	Positive	ND	Contaminated
PCR from eschar	Positive	Positive	Positive

*All patients were treated with doxycycline. NA, not available; ND, not done; IFA, immunofluorescence assay. †From tick bite to symptom onset. ‡Positive cutoff values, IgM = 32; IgG = 128.

**Figure 1:**
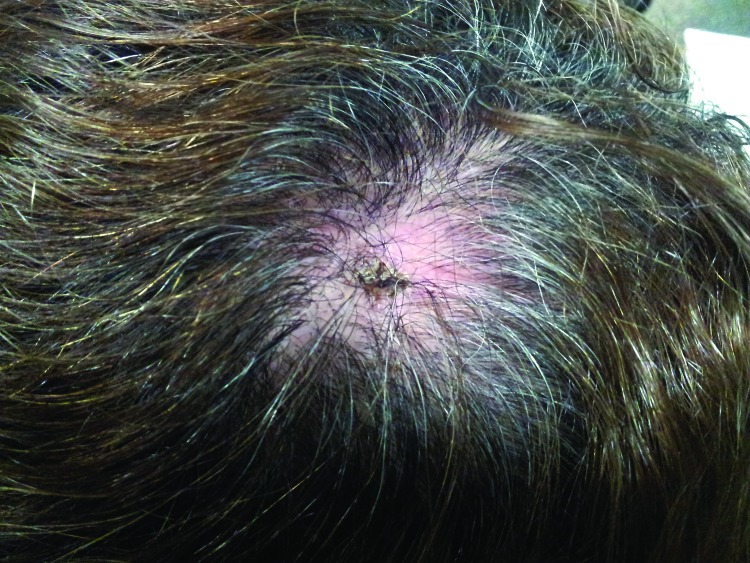
Inoculation eschar surrounded by an erythematous halo at the site of a tick bite on the scalp of a female patient in Portugal. Tick-borne lymphoadenopathy caused by *Rickettsia slovaca* infection was later confirmed.

**Figure 2:**
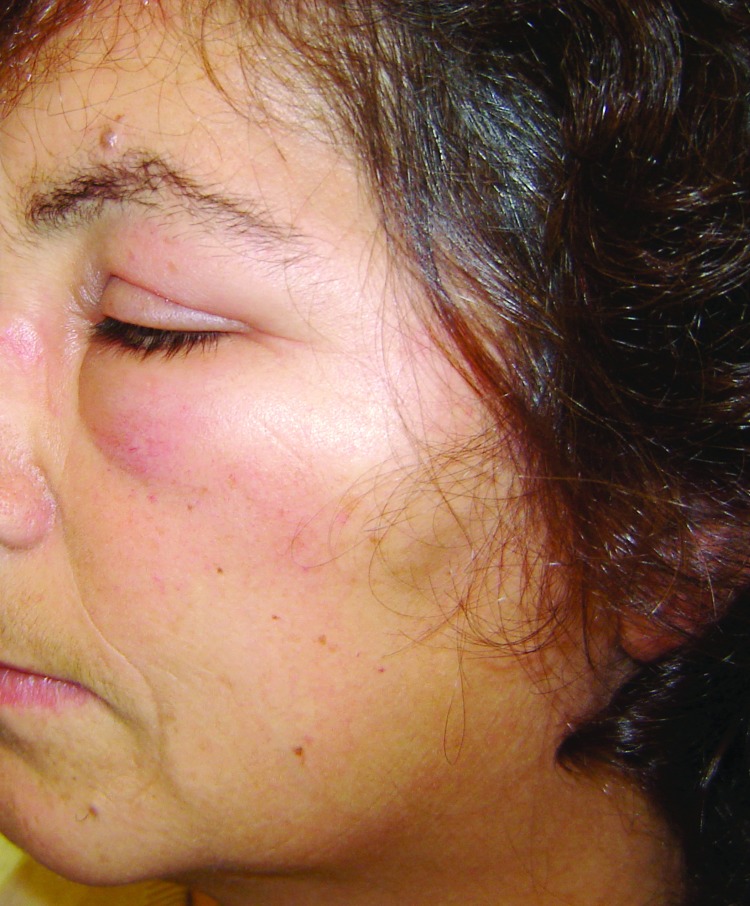
Left peri-orbital edema in a female patient in Portugal. Tick-borne lymphoadenopathy caused by *Rickettsia slovaca* infection was later confirmed.

Physical examination showed a single inoculation eschar surrounded by an erythematous halo on the scalps of all 3 patients; 1 patient later had alopecia develop at the tick bite site (Table; Figure 1). Two patients also had painful cervical and occipital lymphoadenopathies, accompanied by unilateral or bilateral peri-orbital edema (Table; Figure 2). Fever (37.8°C) and a maculopapular rash in the trunk and upper limbs were each visible and reported in 1 patient. 

Laboratory testing showed a slight increase of C-reactive protein in 2 patients and a mild thrombocytopenia in 1, but other results were within normal limits. The patients were treated with doxycycline (200 mg/d) for 7 days with progressive resolution of the clinical signs (e.g., edema, rash).

To confirm the diagnosis of *R. slovaca* infection, serum samples and skin biopsy specimens collected at different times of infection from all patients (Table) were sent to the Portuguese reference laboratory for rickettsioses. Serologic response was analyzed by in-house immunofluorescence assay using *R. slovaca* PoTi443 strain as antigen, as described (*12*). Seroconversion in 2 consecutive samples was demonstrated in patients 1 and 2 by the appearance or increasing levels of IgM and IgG against rickettsiae (Table). Patient 3 had only 1 acute serum sample; test results showed titers for IgM were 32 and for IgG were 64. 

Molecular detection of rickettsial DNA on eschar biopsies was performed by PCR, as described (*13*). Briefly, DNA was extracted from each skin biopsy sample by using a DNeasy Tissue Kit (QIAGEN, Hilden, Germany), according to the manufacturer’s instructions. Nested PCR amplification targeting *omp*A (190-kDa protein) and citrate synthase (*glt*A) fragment genes of *Rickettsia* spp. and sequencing of positive products were done as described (*13*). The sequences were edited by using Lasergene software (DNASTAR, Madison, WI, USA). BLAST analysis (http://blast.ncbi.nlm.nih.gov) showed 100% homology with *glt*A (382/382 bp) and *omp*A (323/323 bp) of *R. slovaca* isolate PoTi443 (GenBank accession nos. HM149281 and HM149286), which was detected in *D. marginatus* ticks in Portugal (*11*).

Skin samples from patients 1 and 3 were used for rickettsial isolation attempts by using Vero E6 cell line and shell-vial technique as described (*14*). (The sample from patient 2 was too small to use for both tests.) After 8 days’ incubation at 32°C, growth of *Rickettsia* spp. was detected and visualized by Gimenez staining and imunofluorescence assay. Positive culture was confirmed for patient 1; however, the culture from patient 3 was contaminated. The *Rickettsia* spp. isolate was characterized by PCR and sequencing as described above for molecular detection. This analysis provided definitive confirmation of the isolate as *R. slovaca.*

## Conclusions

We report 3 confirmed cases of TIBOLA in Portuguese patients, an indication of the emergence of this rickettsial disease in Portugal. The patients were all women, which is in accordance with previous findings of a higher risk for infection for women and children (*4*). Our patients also each showed a tick bite on the scalp associated with the enlargement of lymph nodes, as described in other clinical reports (*3*,*4*–*7*,*9*,*15*). 

Two (67%) of the patients we describe showed facial edema, which is notably higher than Parola et al. reported in his series of patients, where facial edema occurred in 6 (19%) of 49 patients (*15*). Although our number of patients was very small, this sign, associated with the eschar on the scalps of patients 1 and 2, is what led clinicians to further investigate which species of *Rickettsia* was involved in these infections. One of the patients showed residual alopecia, but no patients reported persistent fatigue; these have been described in other patients as frequent complaints in the convalescent stage of disease (*3*,*5*). Low-grade fever (37.8°C) and maculopapular rash each occurred in 1 patient, similar to rates in previous reports for fever (12%–67%) and rash (14%–23%) (*3*,*4*,*15*).

Aside from the typical manifestations of TIBOLA in these patients, isolation and PCR detection followed by genetic characterization of isolates were essential to confirm *R. slovaca* infection. Although the patients showed detectable antibodies against *R. slovaca*, diagnosis on the basis of serologic results only does not distinguish among various SFG rickettsiae, and in Portugal, different Rickettsia spp. can circulate during the same time of year (*13*,*14*). The onset of symptoms (media incubation time 5.5 days) in these patients was in October, April, and May, timing that is associated with seasonal activity of *Dermacentor* spp. ticks. Prevalence rates of *R. slovaca* in these ticks in Europe range from 21% in Hungary to 40.6% in Spain and 41.5% in Portugal (*11*). Based on the similar prevalence of *R. slovaca* in ticks in Portugal and Spain, and in comparison with the large number of patients in Spain with *R. slovaca* infection, it is possible that cases of *R. slovaca* infection in Portugal are not being recognized by clinicians or are being misdiagnosed as Mediterranean spotted fever. 

Although *R. slovaca* is the main etiologic agent associated with TIBOLA, recent studies have indicated that patients with the same characteristic clinical signs may be infected with other *Rickettsia* species, such as *R. rioja* or *R. raoultii* (*4*,*11*), which are also transmitted by *Dermacentor* spp. ticks. Oteo et al. reported that, in Spain, *R. rioja* was the causative agent for almost half of patients with TIBOLA (*4*). In Portugal, a high prevalence (58.5%) of *R. raoultii* has found in *Dermacentor* spp., but this rickettsial species has not been detected in humans (*11*). Because clinical signs can overlap in different rickettsial infections and serologic testing cannot distinguish among SFG rickettsiae, molecular characterization is essential to clarifying the epidemiology of these rickettsial infections.
